# Public health guidance on cardiovascular benefits and risks related to fish consumption

**DOI:** 10.1186/1476-069X-6-31

**Published:** 2007-10-23

**Authors:** Alan H Stern

**Affiliations:** 1 Division of Science, Research and Technology, New Jersey Department of Environmental Protection, 401 E. State St., Trenton, NJ 08625, USA; 2 Department of Environmental and Occupational Health, University of Medicine and Dentistry of New Jersey – School of Public Health, 683 Hoes Lane West, P.O. Box 9, Piscataway, NJ 08854, USA

## Abstract

Historically, concerns with fish consumption have addressed risks from contaminants (e.g., methylmercury (MeHg), and PCBs). More recently public health concerns have widened in appreciation of the specific benefits of fish consumption such as those arising from polyunsaturated fatty acids (PUFAs) in fish oil. Fish contains varying levels of PUFAs and MeHg. Since both address the same health outcomes (in opposite directions) and occur together in fish, great care must be exercised in providing public health guidance. Mozaffarian and Rimm in a recent article (JAMA. 2006, **296**:1885–99) have made a strong case for the beneficial effects of PUFAs in reducing the risk of coronary heart disease, but at the same time, have also broadly discounted the increased risks of coronary heart disease posed by MeHg in fish, stating that "... among adults... the benefits of fish intake exceed the potential risks." This conclusion appears to be based on an inaccurate and insufficiently critical analysis of the literature. This literature is re-examined in light of their conclusions, and the available and appropriate public health options are considered.

## Background

During the past 15 years or so, public health concerns regarding fish consumption have tended to focus mostly on the risks associated with contaminants such as methylmercury (MeHg) and PCBs in fish. More recently, recognition of the general and specific nutritional benefits provided by fish, particularly polyunsaturated fatty acids (PUFA, omega-3 fatty acids (n-3 fatty acids) has appropriately widened the public health focus to include the public health benefits of fish consumption. The potential for both risks and benefits arising from the same food source begs for an overall assessment and ultimately a balancing of risks and benefits in public health guidance. This is all the more so because the major potential health risks of concern, neurodevelopmental effects and cardiovascular effects are precisely the areas where the potential benefits may also occur. This unusual state of affairs means that great care must be exercised in providing public health guidance. It also places a considerable burden on those who would advocate significant changes in existing guidance. In their relatively recent review paper in JAMA [1], Mozaffarian and Rimm make a strong case for beneficial effects of fish-based polyunsaturated fatty acids (PUFA) *per se*, particularly with respect to their apparent reduction in the risk of coronary heart disease. However, I believe that they did not give adequate consideration to the increased risks of coronary heart disease posed by the MeHg in fish, and their broad conclusion that "... among adults... the benefits of fish intake exceed the potential risks" therefore constitutes inappropriate and potentially misleading public health guidance.

## Discussion

**Figure 1:**
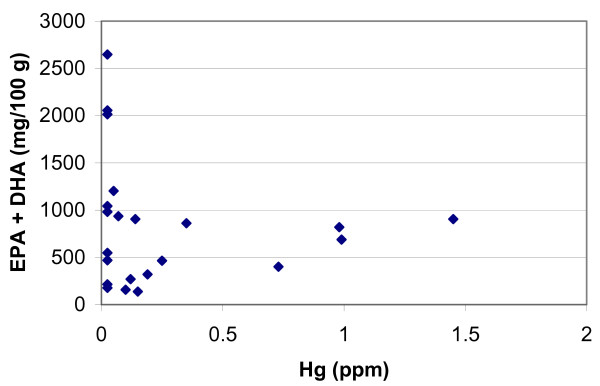
**Mercury and PUFA (EPA+DHA) in commonly consumed marine fish**. Based on Table 2 of Mozaffarian and Rimm [1] (excluding catfish and trout, which are not marine fish). The graph is the author's rendition.

The case that Mozaffarian and Rimm present for the beneficial effects of PUFAs in providing protection against coronary heart disease is seen most strongly in the studies represented in Figs. 1 and 2 of their paper. However, their analysis addresses only part of the public health issues connected with fish consumption. A closer analysis of their data raises serious questions about whether their analysis of the cardiovascular risks and benefits of fish consumption take both PUFAs *and *methylmercury into account as opposed to merely addressing PUFA intake in isolation. Many of the data in the studies they analyze reflect studies in which subjects consumed purified fish oil. To the extent that some of these studies, in fact, reflect fish intake, it is not clear that they also reflect significant MeHg intake. Higher levels of PUFA intake (in studies of fish consumption) do not necessarily reflect increased fish intake and, by extension, do not necessarily imply higher levels of MeHg intake, but may simply reflect, instead, intake of fish species with higher PUFA content. Oily fish (i.e., those high in PUFAs) are not characteristically also high in Hg. This can be seen in the data for commonly consumed species of fish presented in Table 2 of the Mozaffarian and Rimm paper and shown here (minus catfish and trout, which are not ocean fish) in Figure 1 (author's rendition).

This figure shows that fish with high PUFA content are not particularly high in Hg, and the fish with the highest Hg concentration (shark, swordfish, tilefish) do not have particularly high levels of PUFA. It is also worth noting that these particular species of fish do not account for a significant portion of national fish consumption [2]. Thus, it is likely that for studies analyzed by Mozaffarian and Rimm that quantified PUFA intake on the basis of fish consumption, MeHg intake was, on average, low, especially for that portion of the cohort with high PUFA intakes. Thus, these observations probably do not tell us much about the cardiovascular risk for those fish consumers with elevated MeHg intakes and moderate or even high PUFA intakes. Without focusing on the subset of the population with elevated MeHg intakes, or without applying a case-control approach and starting with those who experienced coronary events, we don't know much about the *interaction *of PUFAs and MeHg. This can only be done in studies that examine the association of both PUFAs and MeHg with cardiovascular risk.

The number of studies that do examine both the PUFAs and MeHg in relation to cardiovascular outcome is relatively small. In their paper, the authors state: "Several studies have evaluated the relationship between mercury exposure and incidence of cardiovascular disease. The conflicting results provide inconclusive evidence for cardiovascular toxicity of mercury. Notably, in the two studies observing higher risk with higher mercury levels, the net effect of fish consumption was still beneficial: greater mercury exposure lessened the benefit associated with consumption of fish or n-3 PUFAs, but did not increase overall risk." The studies cited in support of this statement are clearly not all of equal quality or applicability. Of the five studies cited [3-7], two, Hallgren et al. [3] and Ahlqwist et al. [3], are not comparable to the others. The Hallgren et al. study included only 78 MI cases compared to 684 in the Guallar et al. study [5], 470 CHD cases in the Yoshizawa et al. study [5], and 282 acute coronary event cases plus 132 cardiovascular disease cases in the Virtanen et al. study [6]. In the high-Hg – low-PUFA group in Hallgren et al., there were only 4 cases. Thus, the Hallgren et al. study had relatively less power to see either MeHg effects per se, or to evaluate the interaction of PUFAs and MeHg. Hallgren et al. used MeHg and PUFA exposure data that were collected up to 10 years prior to the case identifications. This increases the potential for exposure misclassification and makes identification of a true association less likely. Furthermore, the Hallgren et al., and Ahllqwist et al. studies were the only studies under consideration that included women. The Ahlqwist et al. study is particularly problematic for assessing the association of MeHg, PUFA and cardiovascular outcomes. The study involved only women. Hg concentration was determined in serum only, and thus, as the authors of that study note, disproportionally reflects inorganic Hg exposure (e.g., from dental amalgams) rather than MeHg exposure. Neither Hg concentrations, nor fish consumption data are actually reported. Furthermore, for most subjects, the Hg exposure data were collected up to 25 years prior to case identification. Thus, of the five studies cited by Mozaffarian and Rimm as addressing the association between Hg exposure and cardiovascular risk, two are positive, and of the three negative studies, two are not high quality studies for this purpose and should not have been considered. The remaining negative study, Yoshizawa et al. [7], is a high quality study. However, as the authors of that paper discuss, the results are potentially confounded by the presence of the dentists among the health professionals. The dentists constitute the majority of the subjects, and their exposure to elemental Hg dominates their overall Hg exposure. The Yoshizawa et al. study can reasonably be considered equivocal with respect to MeHg expsosure *per se*. Thus, Mozaffarian and Rimm misstate the case in stating that "the conflicting results provide inconclusive evidence for cardiovascular toxicity of mercury" While I would agree that the positive evidence is not completely conclusive, the negative evidence is weak, Furthermore, the two positive studies, Guallar et al. and Virtanen et al., are, in fact, more persuasive than might be inferred from a simple count of citations. The Guallar et al. study is a large multi-center study that incorporates data from geographically diverse communities with different types of fish consumption, different levels of MeHg and PUFA intake, different overall diets and different lifestyle characteristics. No one center dominated the results, and elimination of the two centers with the highest MeHg exposure did not alter the findings. In addition, in the Guallar study, Hg exposure data (toenails) were collected in close proximity to the first myocardial infarction. Exposure data are therefore closely temporally linked to the outcome. The Virtanen et al. study is a prospective study with a study cohort of 1,871. This study represents an analysis of an extended follow-up period for a cohort in which a relationship between MeHg exposure and acute coronary events had been previously been observed [8,9]. MeHg exposure was estimated from hair Hg concentration, a well-validated biomarker for MeHg exposure. Based on the data provided for average follow-up time and the dates of hair sampling, hair samples for Hg were obtained at about the mid-point of the entire range of the follow-up period, and most of the samples appear to have been taken within 4–5 years of an acute coronary event. The findings in this cohort have been consistent across studies and follow-up periods.

Even more critical, however, from the standpoint of influencing public health policy is the assertion by Mozaffarian and Rimm that: "Notably, in the two studies observing higher risk with higher mercury levels [Guallar et al., and Virtanen et al.], the net effect of fish consumption was still beneficial: greater mercury exposure lessened the benefit associated with consumption of fish n-3 PUFAs, but did not increase overall risk." I can find no basis of support for that statement. Rather, in their Table 4, Guallar et al. show that without adjustment for DHA (i.e., when both Hg and DHA exposure are considered together), the odds-ratio for MI increases dramatically in the highest quintile of Hg exposure (1.0–1.47, p for trend = 0.01). While without adjustment for Hg (again considering Hg and DHA exposure together), the odds-ratio for MI does not differ across the quintiles of DHA (1.0-0.8, p for trend = 0.23). That is, DHA is not protective against the increased risk of MI due to Hg exposure. A downward trend in the odds-ratio with increasing DHA is only seen *after *controlling for Hg exposure. That is, only when the effect of Hg is held constant across levels of DHA exposure is an *underlying *protective effect of DHA seen. Likewise, in Tables 2 and 3 of Virtanen et al., the authors report that without adjustment for DHA+DPA (i.e., considering both the PUFAs and Hg), the relative risk for acute coronary events in men in the upper third of Hg exposure is increased 55–60% compared to men in the lowest third of exposure. Furthermore, the relative risk of acute coronary events with increasing DHA+DPA does not extend below 1.0 when hair Hg concentration exceeds 2.03 ppm (the upper third of the distribution of hair Hg concentration in the study population). These results show that in this population, when Hg intake was moderately elevated, Hg increased the risk of an acute coronary event and that risk was not offset by the PUFA intake.

The broad conclusion of Mozaffarian and Rimm that "... among adults... the benefits of fish intake exceed the potential risks," appears to rest on the assertions that there is no clear evidence for adverse effects of MeHg at current levels of intake from fish consumption, and that any increase in risk that might be present from MeHg intake from fish consumption is more than offset by the concurrent PUFA intake. Based on the foregoing, I am at a loss to see how the data support such an unequivocal statement. The results from both Virtanen et al., and Guallar et al. show a clear increase the risk of acute coronary events with moderately increased MeHg intake. Furthermore, Virtanen et al. in their Table 3, present evidence for an interaction between the beneficial effects of PUFA intake and the risks from MeHg intake (relative risk of acute coronary event per unit increase in DHA+DPA stratified by low and high hair Hg = 0.69 and 1.06 respectively, p value for interaction = 0.023), such that at moderate levels of MeHg intake, not only does MeHg risk increase, but PUFA benefit decreases, with the result that the risk outweigh the benefits. This is by no means to say that people should avoid fish. Eating fish, or not eating fish, is not the relevant choice, and not the appropriate public health issue. Rather, the data presented by Mozaffarian and Rimm in their Table 2 shows there is a variety of easily available fish that offers high PUFAs and low MeHg. In fact, even if one considers fish consumption at the 12 ounces per week (340 g/week) recommended by the U.S.FDA specifically for women of childbearing age on the basis of MeHg developmental risk to the fetus [10] rather than at the likely less restrictive rate that would be appropriate for adult men, about half the commonly consumed fish and shellfish in Mozaffarian and Rimm's Table 2 would meet the criterion of providing the 250 mg/day of EPA and DHA they identify as the target to reduce coronary heart disease *and *having Hg concentrations characterized by FDA as suitable for regular consumption. This pattern is also seen in the more comprehensive database presented by Mahaffey [11]. It is also worth noting in this context that there are other dietary sources of omega-3 PUFAs such as flaxseed oil, canola oil, and soybean oil as well as refined fish oil [12]. While these may provide alternatives for those who are not amenable to regular consumption of fish, I believe that the overall nutritional benefits of fish make consumption of high PUFA-low Hg fish the most desirable option at the present time.

## Conclusion

That there are easily available fish that offer both high PUFA and low MeHg and that consumers should choose wisely among the available fish so as to maximize the benefit and decrease the risks is, I believe, the appropriate public health message. The overly broad and thus unsupportable statement by Mozzaffarian and Rimm that "... among adults... the benefits of fish intake exceed the potential risks" is, given the available data, an inappropriate public health message.

## Competing interests

The author(s) declare that they have no competing interests.

## Authors' contributions

AHS is the sole author and is responsible for the entire manuscript. The work does not necessarily reflect the policies of the New Jersey Department of Environmental Protection.

## References

[B1] Mozaffarian D, Rimm EB (2006). Fish intake, contaminants, and human health: evaluating the risks and the benefits. JAMA.

[B2] Nesheim MC, Yaktine AL, Institute of Medicine (2007). Seafood Choices – Balancing Benefits and Risk.

[B3] Hallgren CG, Hallmans G, Jansson JH, Marklund SL, Huhtasaari F, Schutz A, Stromberg U, Vessby B, Skerfving S (2001). Markers of high fish intake are associated with decreased risk of a first myocardial infarction. Br J Nutr.

[B4] Ahlqwist M, Bengtsson C, Lapidus L, Gergdahl IA, Schutz A (1999). Serum mercury concentration in relation to survival, symptoms, and diseases: results from the prospective population study of women in Gothenburg, Sweden. Acta Odontol Scand.

[B5] Guallar E, Sanz-Gallardo MI, van't Veer P, Bode P, Aro A, Gomez-Aracena J, Kark JD, Riemersma RA, Martin-Moreno JM, Kok FJ (2002). Heavy Metals and Myocardial Infarction Study Group. Mercury, fish oils, and the risk of myocardial infarction. N Engl J Med.

[B6] Yoshizawa K, Rimm EB, Morris JS, Spate VL, Hsieh CC, Spiegelman D, Stampfer MJ, Willett WC (2002). Mercury and the risk of coronary heart disease in men. N Engl J Med.

[B7] Virtanen JK, Voutilainen S, Rissanen TH, Mursu J, Tuomainen TP, Korhonen MJ, Valkonen VP, Seppanen K, Laukkanen JA, Salonen JT (2005). Mercury, fish oils, and risk of acute coronary events and cardiovascular disease, coronary heart disease, and all-cause mortality in men in eastern Finland. Arterioscler Thromb Vasc Biol.

[B8] Salonen JT, Seppanen K, Nyyssonen K, Korpela H, Kauhanen J, Kantola M, Tuomilehto J, Esterbauer H, Tatzber F, Salonen R (1995). Intake of mercury from fish, lipid peroxidation, and the risk of myocardial infarction and coronary, cardiovascular, and any death in eastern Finnish men. Circulation.

[B9] Rissanen T, Voutilainen S, Nyyssonen K, Lakka TA, Salonen JT (2000). Fish oil-derived fatty acids, docosahexaenoic acid and docosapentaenoic acid, and the risk of acute coronary events: the Kuopio ischaemic heart disease risk factor study. Circulation.

[B10] What You Need to Know About Mercury in Fish and Shellfish 2004 EPA and FDA Advice For: Women Who Might Become Pregnant Women Who are Pregnant Nursing Mothers Young Children. http://www.cfsan.fda.gov/~dms/admehg3.html.

[B11] Mahaffey KR (2004). Fish and shellfish as dietary sources of methylmercury and the omega-3 fatty acids, eicosahexaenoic acid and docosahexaenoic acid: risks and benefits. Environ Res.

[B12] Psota TL, Gebauer SK, Kris-Etherton P (2006). Dietary omega-3 fatty acid intake and cardiovascular risk. Am J Cardiol.

